# Long-lasting ergot lipids as new biomarkers for assessing the presence of cereals and cereal products in archaeological vessels

**DOI:** 10.1038/s41598-018-22140-z

**Published:** 2018-03-02

**Authors:** Jeannette J. Lucejko, Jacopo La Nasa, Francesca Porta, Alessandro Vanzetti, Giuseppa Tanda, Claudio Filippo Mangiaracina, Alessandro Corretti, Maria Perla Colombini, Erika Ribechini

**Affiliations:** 1 Dipartimento di Chimica e Chimica Industriale, Università di Pisa, Via G. Moruzzi 13, 56124 Pisa, Italy; 2 Dipartimento di Scienze dell’Antichità, Università degli Studi di Roma “La Sapienza”, Piazzale Aldo Moro, 5, 00185 Roma, Italy; 3 Dipartimento di Storia, Beni Culturali e Territorio, Università degli Studi di Cagliari, Piazza Arsenale 1, 09124 Cagliari, Italy; 4 LA3M, Aix Marseille Université, CNRS, rue du château de l’Horloge 5, 13094 Aix-en-Provence, France; 5 Laboratorio di Storia, Archeologia, Epigrafia, Tradizione dell’antico, Scuola Normale Superione, Piazza dei Cavalieri, 56126 Pisa, Italy

## Abstract

Cereals were very important in ancient diets, however evidence from archaeological sites of the vessels used for processing or storing cereals is comparatively rare. Micro-organisms, as well as chemical-physical effects can easily degrade cereals during the burial period. This can lead to a complete cereal decay and to serious difficulties in estimating the intensity of use of the cereals by ancient populations. Here, we present a novel biomarker approach entailing the detection of secondary lipid metabolites produced by ergot fungi (genus *Claviceps*), which are common cereal pests. The aim was to identify the original presence of *Gramineae* and to indirectly establish if vessels were used for cereal storage/processing. The fatty acid and TAG-estolide profiles of the remains from more than 30 archaeological vessels were investigated by gas chromatography/mass spectrometry (GC/MS) and high performance liquid chromatography/high resolution mass spectrometry (HPLC/ESI-Q-ToF). The detection of lipids derived from ergot in archaeological and historic contexts rests on its complex chemistry, providing a unique and relatively recalcitrant chemical signature for cereals. This research demonstrated that the combination of our innovative biomarker approach along with environmental and archaeological evidence can provide unprecedented insights into the incidence of cereals and related processing activities in ancient societies.

## Introduction

Although cereals played a key role in ancient times, e.g. in the Neolithic diet, their botanical and chemical remains are relatively rare at archaeological sites. When cereals are morphologically preserved or even found as charred remains, the analysis of the DNA, phytoliths as well as mass spectrometric analysis of the organic remains can lead to interesting conclusions regarding the artefact functions and cereals diffusion^1–4^. Cereals are lignocellulosic substrates and thus prone to attack by insects, bacteria or other micro-organisms, as well as degradation by chemical-physical processes occurring during the burial period^5^. Charred cereals are comparatively less prone to microbial degradation, and are thus more easily found at archaeological sites. Some attempts to determine the presence of cereals from the analysis of charred remains have been reported in the literature^6–9^. However, understanding the importance of cereals in ancient times remains a difficult and unexplored task.

**Figure 1:**
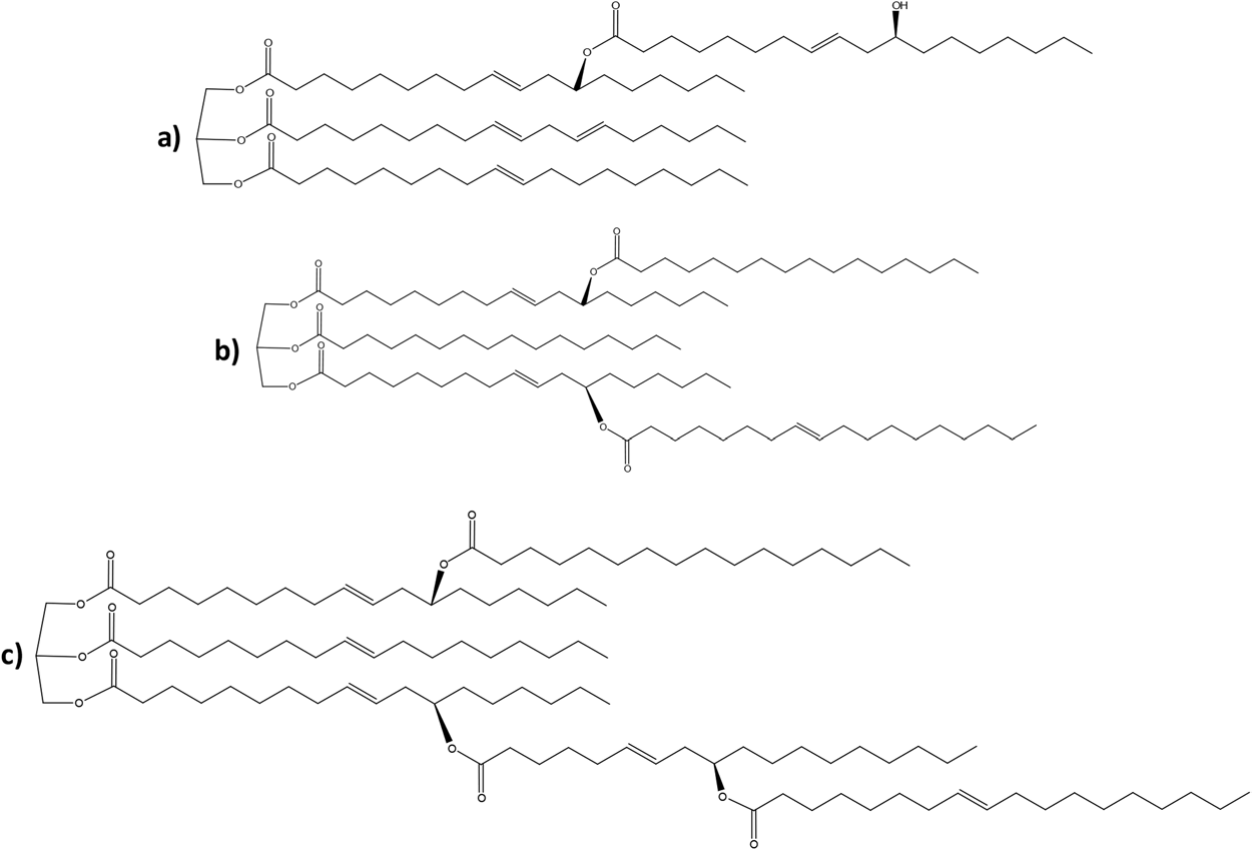
Typical estolides species detected in the ergot acylglycerides fraction characterized by four (**a**), five (**b**), and six (**c**) acyl chains.

**Figure 2:**
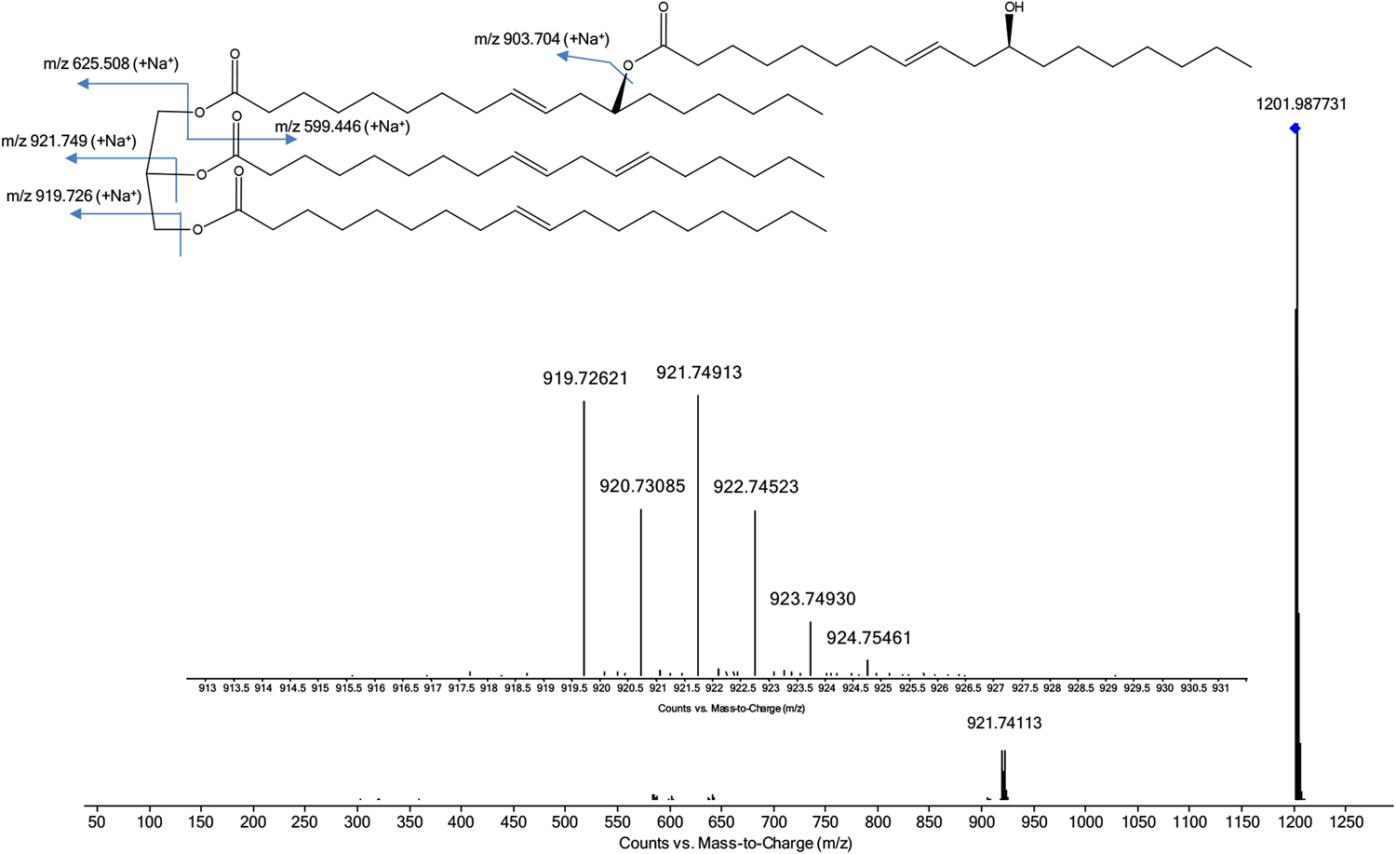
High resolution mass spectra and fragmentation pattern of RnRnOP sodiate adduct (estolides with 4 acyl chains). Acyl substitutes are named according to the following fatty acid abbreviations: Rn: ricinoleyl, O: oleyl (C_18:1_). P: palmityl (C_16:0_).

This paper presents an innovative biomarker approach entailing the recognition of the exclusive lipids biosynthesized by ergot fungi of the genus *Claviceps*. *Claviceps* fungi (ergot) are common pests of *Gramineae*^10^, and they metabolically produce lipids characterized by a complex mixture of more than 70 compounds, consisting of a series of diglycerides, triglycerides and high molecular weight estolides, with ricinoleic acid (12-hydroxy-9-octadecenoic acid) being the most abundant acyl substituent^11^. Estolides (Fig. 1) result from secondary acylation reactions, where additional fatty acids are esterified to the hydroxyl moieties of ricinoleic acid, leading to the formation of high molecular weight species^12^. The use of high resolution tandem mass spectrometry allowed us to perform a structural characterization of these species, as reported in Fig. 2.

Lipids from the archaeological vessels were extracted and analysed using well-established protocols^13–17^. The amount of ceramic or stone required for the analysis was less than 1 g, ensuring to perform the analysis without destroying or sensibly damaging the archaeological artefacts.

Gas chromatography/mass spectrometry (GC/MS) following alkaline hydrolysis, solvent extraction and derivatization, was used as a preliminary screening approach to characterise the fatty acid profiles of the archaeological samples. Liquid chromatography coupled with high resolution tandem mass spectrometry (HPLC/ESI-Q-ToF) was then used to further investigate the lipid fraction of those samples that showed evidence of ricinoleic acid in the fatty acid profile.

Our aim was to obtain a comprehensive and detailed picture of acyl-glyceride composition of such samples and to identify traces of ergot. To maximize the extraction of lipid residues from the archaeological artefacts, the samples were subjected to a novel microwave-assisted extraction. Given that this extraction approach is highly efficient means that smaller samples can be used. It is also more efficient than using a traditional ultrasonic bath, although the material is polymerized or oxidized as expected with archaeological lipids^16,18^.

## Results and Discussion

Adopting this lipid profiling approach, we studied the residues from 32 vessels: 15 potsherds recovered from two archaeological sites, in Sardinia and Calabria, and 17 stone vessel fragments from excavations in Sicily, in Italy.

The ceramic samples from Sardinia were recovered from the anteroom and from the dromos of the Domus de Janas IV, in the necropolis of Molia (Illorai). The ceramic samples were characterized by three different shapes (olla, bowl and pan) which were dated to the last Neolithic period (3970-3340 BC). Only animal remains were identified at the site, indicating that the dromos and the anteroom were probably used as the place for ritual offerings^19^.

**Figure 3:**
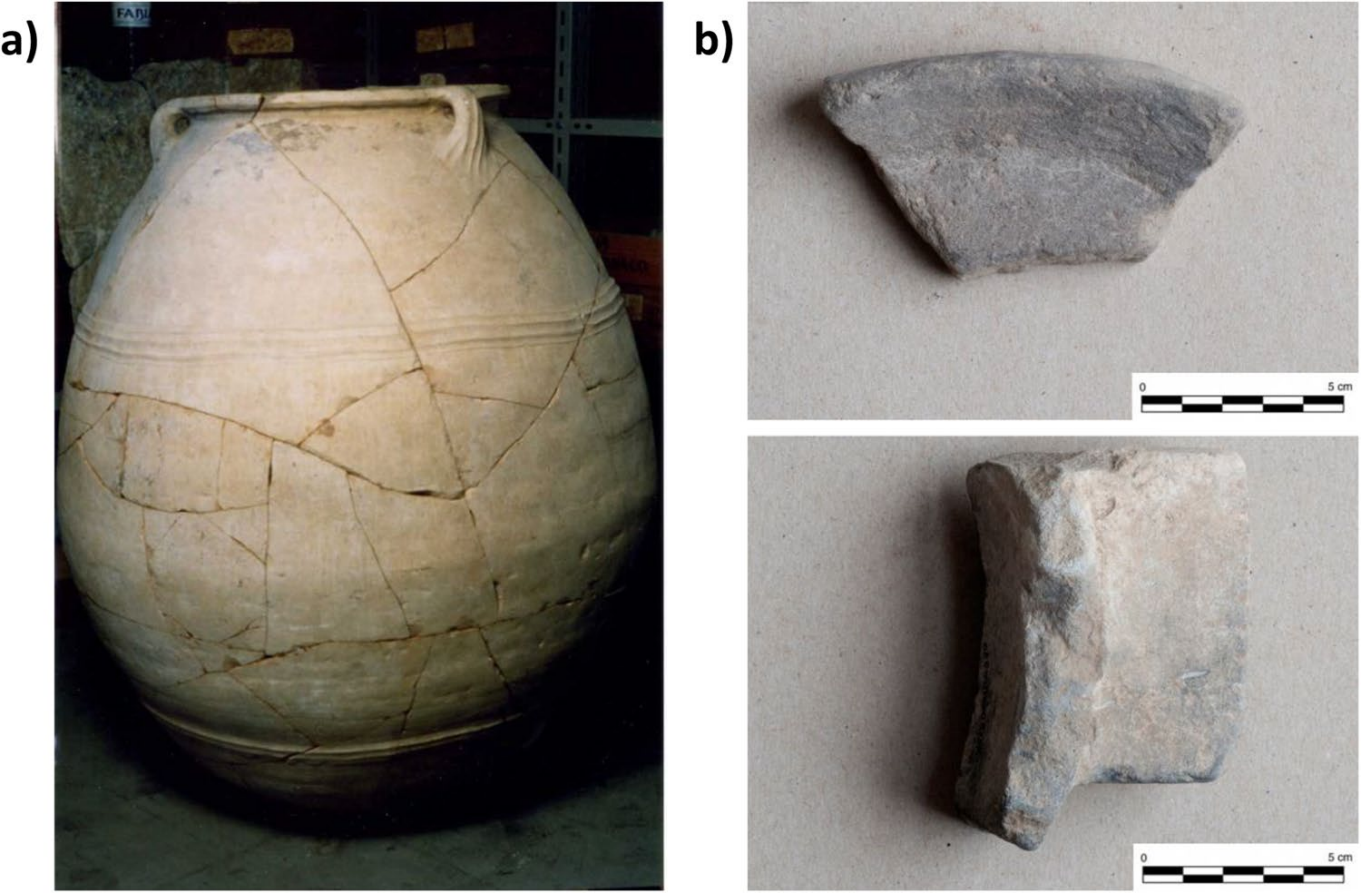
(**a**) Pithoi recovered from the protohistoric site of Broglio di Trebisacce (Calabria); (**b**) stone vessels fragments from the site of Entella and Palermo (Sicily).

The ceramic fragments from Calabria came from vases (pithoi) recovered from the protohistoric site of Broglio di Trebisacce (Cosenza). These pithoi (Fig. 3) could contain up to 1000 liters of foodstuff. They were first produced as a result of advanced technologies from the Aegean world, where they were used in palaces and private storerooms during the Middle and Late Minoan/Helladic periods (ca. 1.950-1.100 BC)^20^, and later. The Broglio pithos sherds date to the Late Bronze Age in Italy (LBA: 1.300-950 BC) and are decorated similarly Cretan and Cypriot vases^21^.

The stone vessels from Sicily (Fig. 3) came from Entella and Palermo. They belong to a class of soft-stone artifacts from the Islamic Age (827–1061 AD) to the Age of Frederick II, king of Sicily (1194-1250 AD). Based on their morphology, the soft-stone vessels can be classified into three forms. The first is a rectangular vessel with vertical walls (approx. 3 cm) and four feet. The second form is a flat circular disk with rounded edges. This artifact shows burn marks on both surfaces and was probably a lid or an oven plate/base. The third is a broad, circular dish (diam. approx. 25–28 cm) with a low vertical wall (approx. 1.5 cm) which is triangular in section. The internal surface of these vessels shows traces of fire blackening. Due to the morphological resemblance to the ceramic *testelli* which were used for cooking flour-based foods, e.g. bread, particularly in the Tyrrhenian area, soft-stone vessels belonging to this last shape are also likely to be *testelli*^22^.

Out of the 32 vessels analysed by GC/MS, ten vessels (five from Sardinia: Illo_O3, Illo_O4, Illo_O8, Illo_O9, Illo_O10; four from Calabria: T810, T8548, T8824, Dolium A, and one from Sicily: P1) exhibited the presence of ricinoleic acid and other common features highlighted by monocarboxylic fatty acids such as palmitic, oleic and stearic acids and dicarboxylic fatty acids such as azelaic acid. In addition to fatty acids, some samples also showed the presence of other organic compounds. In Dolium T810 from Calabria and in Illo_O8 from Sardinia, tricyclic diterpenoid acids with an abietane skeleton were found with 7-oxo-dehydroabietic and dehydroabietic acids being the main ones, together with di-dehydroabietic acid as well as other oxidised (7-oxo-didehydroabietic acid, 15-hydroxy-7-oxo-dehydroabietic acid, 15-hydroxy-7-oxo-dehydroabietic acid) and methylated (methyl-dehydroabietate and methyl-7-oxo-dehydroabietate) forms. These data indicate the occurrence of pine pitch^23,24^.The GC/MS profile of the P1 sample was also characterized by the presence of 18-norabietane and other phenanthrene derivatives, as well as dehydroabietic acid. This stone vessel was thus probably subjected to a heating process, as also suggested by the black burning traces on the external part of the vessel.

The lipid fractions extracted from the 10 vessels that showed evidence of ricinoleic acid in the fatty acid profile were further investigated by HPLC/ESI-Q-ToF.

The lipid profile comparison of the samples with those previously published for ergot^11^ showed an unequivocal correlation, with more than 50 species that could be related to fungal activity.

**Figure 4:**
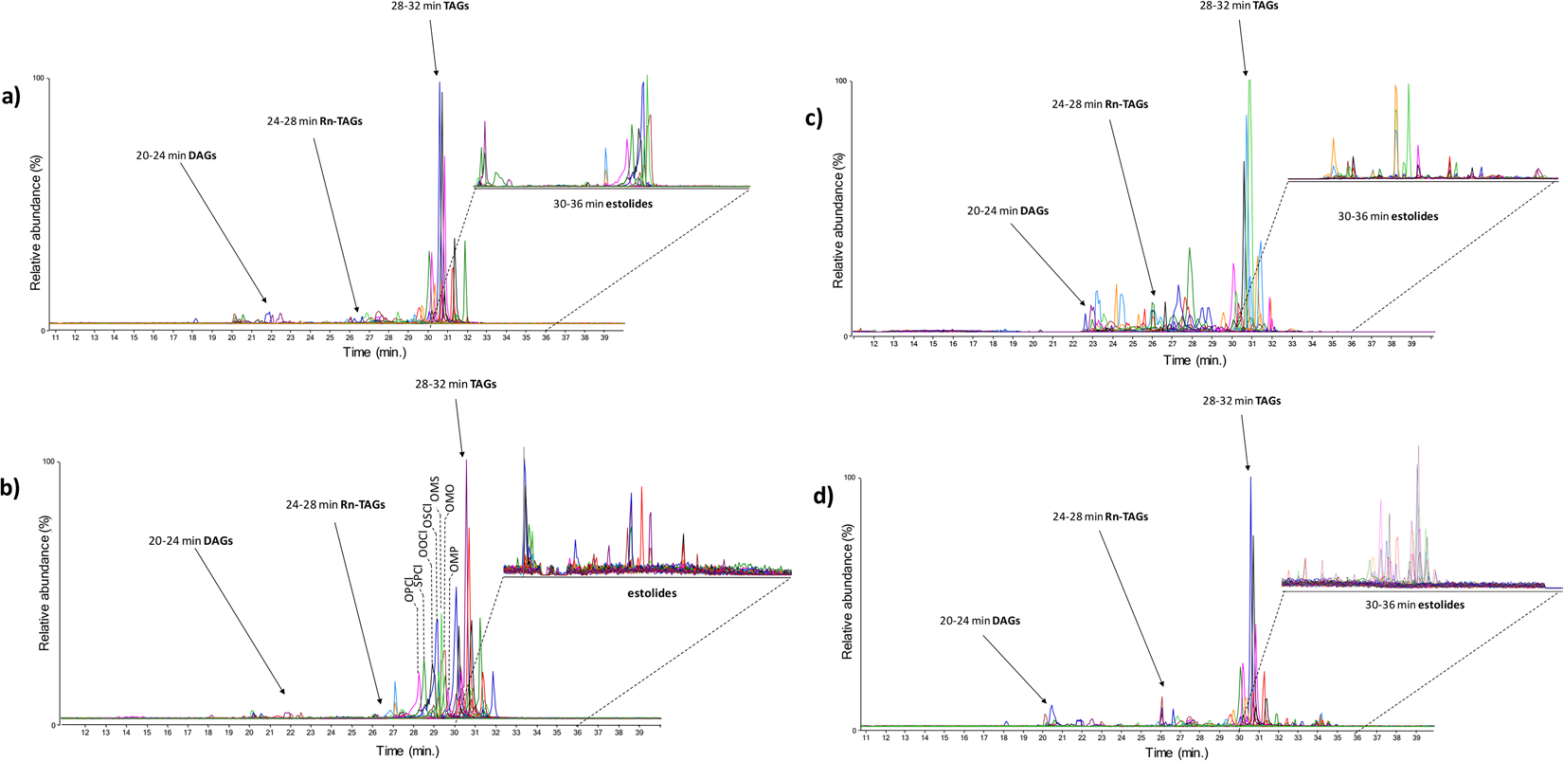
HPLC/ESI-Q-ToF chromatograms of the triglycerides fraction of samples P1 (**a**), Illo_O8 (**b**), Dolium A (**c**), and Dolium T810 (**d**) the chromatograms were obtained by overlapping the extract ion chromatograms of 56, 92, 74 and 74 ergot acylglycerides, respectively. In the chromatogram obtained for Illo_O8 are reported the triglycerides not related to the ergot activity. Triglycerides are named according to the following fatty acid abbreviations: Rn: ricinoleyl, O: oleyl (C_18:1_), S: stearyl (C_18:0_), P: palmityl (C_16:0_), P: palmitoleyl (C_16:1_), M: myristyl (C_14:0_), and Cl: caprilyl (C_8_).

Figure 4 shows the chromatograms obtained by HPLC/MS for Dolium A, Dolium T810, Illo_O8, and P1 samples. Besides the common features, the various samples exhibited particular lipid profiles probably due to the different age, burial environments and conservation states. For instance, Dolium A showed a high amount of triglycerides containing ricinoleic acid and a relative low abundance of estolides. At the same time, in Dolium T810 the estolides were completely preserved. Although in archaeological lipids, oxidation and polymerization involving the double bonds of unsaturated acyl substituents can frequently lead to a loss of information on the chemical composition, the low degree of unsaturation of most of the high molecular species in ergot protects them from extensive degradation, which means we can recognise its lipid profile even in very old or degraded samples. Sample P1 is an example of the alteration of the original lipid composition due to anthropogenic manipulation, which entails the exposure of the stone vessel to high temperatures. The lipid profile of this sample was characterized by a higher oxidation degree than the other samples, with TAGs with saturated acyl substituents as the main species, triglycerides characterized by more resistance to heating, and trace levels of estolides. All this further demonstrates that the unique ergot lipids survive quite well in different archaeological vessels and environments, and that ergot TAGs and estolides can be effectively used to assess the original presence of cereals and/or related processing products and activities.

Our approach is also valid when lipid substances other than ergot are present in the same sample as in Illo_O8 from Sardinia. In this case, ergot lipids can be clearly recognised despite the simultaneous presence of triglycerides ascribable to animal fats^25,26^. High resolution tandem mass spectrometry (which is an integral feature of the HPLC/ESI-Q-ToF system) was used to differentiate among isobaric isomers, leading to an unambiguous identification of the triglycerides and estolide species and of their origin^27^.

The information obtained with our analytical approach could be extremely useful for the interpretation of archaeological findings, as can be highlighted by the results obtained on these archaeological samples. In detail, the biomarkers related to ergot activities in the vessels from Sardinia strongly suggest the use of such pottery for ritual offerings containing cereals. The results on the pithoi from Calabria along with the archeological evidence (at Broglio di Trebisacce, a storeroom containing 5 pithoi was discovered) demonstrate an improvement in agricultural techniques and production, as well as in storage capacities. Such pithoi could thus have been used to store cereals as well as flour. Interestingly, our results could also be used to study anthropogenic activities and habits, as shown in the results from the Palermo vessel that suggested that such circular stone vessels were used for cooking flour-based dishes.

To conclude, the joint use of GC/MS and HPLC/ESI-Q-ToF enabled us to detect for the first time more than fifty secondary lipid metabolites produced by ergot (*Claviceps*) in vessels from archaeological and historic contexts. This novel approach demonstrated that the complex and robust chemistry of ergot lipids provides a unique and relatively recalcitrant chemical signature for cereals in archaeological vessels. With our research, we can learn more about how and to what extent cereals were used in ancient societies and what kind of vessels were employed for cereal processing and storage, thereby providing new insights into the life of ancient civilizations.

## Methods

### Chemicals

The GC/MS analyses were performed using, *n-*hexane, diethyl ether, and isooctane (HPLC grade; Sigma-Aldrich, U.S.). The derivatization was performed using N,O-bis(trimethylsilyl)trifluoroacetamide (BSTFA) added with 1% trimethyl-chlorosilane (Sigma-Aldrich). The tridecanoic acid and hexadecane solutions were prepared in isooctane (140 µg/g and 142 µg/g respectively) using standards with a 99% purity (Sigma-Aldrich).

The HPLC analyses were performed using *n*-hexane, *iso*-propanol, chloroform and methanol (HPLC-MS grade; Fluka, U.S.).

### Samples

Ceramic sherds and stone vessels were not washed before sampling and analyses. Washing can remove labile constituents of absorbed residues as well as introduce contaminants. The samples were obtained collecting ceramic fragments and stone vessels (roughly 1 g) using a scalpel and after grinding them to powder to perform GC/MS and HPLC/MS analyses.

### Gas chromatography/mass spectrometry

The GC/MS analyses were performed systematically on samples taken from the internal and external surfaces of each archaeological object and on the soil from the excavation in order to exclude the possibility of contamination. The samples were saponified with a 800 µL of solution of KOH_MeOH_ 10% and KOH_H2O_ 10% (1:3) at 60 °C for 180 min^13^.

After the saponification, all the analytes were extracted using a multi-step extraction with *n-*hexane for the neutral compounds and diethyl ether after acidification with hydrochloric acid for the acidic species.

The two extracts were dried with N_2_ stream and derivatized with 20 µL of BSTFA containing 1% trimethylchlorosilane, 150 µL of isooctane and 5 µL of tridecanoic acid solution at 60 °C for 30 min. 5 µL of hexadecane solution were added before the injection^14^.

GC/MS analyses were performed using an Agilent Technologies 6890 N gas chromatograph with a split/splitless injection port and coupled with a 5973 mass selective single quadrupole mass spectrometer (Agilent Technologies, USA). The separation was performed with a fused silica capillary column HP-5 MS (5% diphenyl-95% dimethyl-polysiloxane, 30 × 0.25 mm, internal diameter, 0.25 μm film thickness, Agilent Technologies). The chromatographic and mass spectrometric conditions were adapted from^15,18,28^. The analytes were identified by comparison with mass spectra libraries (WILEY275, NIST 1.7).

### High performance liquid chromatography/high resolution mass spectrometry

As for the GC/MS approach, the HPLC analyses were performed on samples from the internal and external surfaces of each archaeological object, and on the soil from the excavations in order to rule out the presence of contaminations. The samples were extracted using a Milestone microwaves Ethos One (power 600 W) with a chloroform-hexane (3:2) mixture (300 µL) for 25 min at 80 °C. The extracts were dried, diluted and filtered 0.45 µm PTFE filter (Grace Davison Discovery Sciences, U.S.) before the injection^16^.

HPLC/ESI-Q-ToF analyses were performed using a 1200 Infinity HPLC, coupled with a Quadrupole-Time of Flight tandem mass spectrometer 6530 Infinity Q-ToF detector by a Jet Stream ESI interface (Agilent Technologies).

The separation was obtained with a Poroshell 120 EC-C18 column (2.7 μm particle size, 3.0 × 5.0 mm) with a Zorbax eclipse plus C-18 guard column (5 μm particle size, 4.6 × 12.5 mm); the flow rate was 0.3 mL/min and 1 µL of solution was injected. The separation was thermostatically controlled at 45 °C^29^.

Separation was obtained by using a gradient of methanol/water 85:15 (eluent A) and iso-propanol (eluent B), programmed as follows: 90% A for 5 min, followed by a linear gradient to 90% B in 30 min, then 10 min at 90% B. Re-equilibration time for each analysis was 10 min^30,31^.

The mass spectrometry system conditions were adapted from previous published works^16,18,29,30^, and the data elaboration was performed using MassHunter Workstation Software (B.04.00). The structures of all the identified species were interpreted by their MS^2^ spectra^29,32^.
